# Privacy Preserving Image Encryption with Optimal Deep Transfer Learning Based Accident Severity Classification Model

**DOI:** 10.3390/s23010519

**Published:** 2023-01-03

**Authors:** Uddagiri Sirisha, Bolem Sai Chandana

**Affiliations:** School of Computer Science and Engineering, VIT-AP University, Amaravathi 522237, India

**Keywords:** accident images, privacy preserving, key generation, deep learning, severity classification, hyperparameter tuning

## Abstract

Effective accident management acts as a vital part of emergency and traffic control systems. In such systems, accident data can be collected from different sources (unmanned aerial vehicles, surveillance cameras, on-site people, etc.) and images are considered a major source. Accident site photos and measurements are the most important evidence. Attackers will steal data and breach personal privacy, causing untold costs. The massive number of images commonly employed poses a significant challenge to privacy preservation, and image encryption can be used to accomplish cloud storage and secure image transmission. Automated severity estimation using deep-learning (DL) models becomes essential for effective accident management. Therefore, this article presents a novel Privacy Preserving Image Encryption with Optimal Deep-Learning-based Accident Severity Classification (PPIE-ODLASC) method. The primary objective of the PPIE-ODLASC algorithm is to securely transmit the accident images and classify accident severity into different levels. In the presented PPIE-ODLASC technique, two major processes are involved, namely encryption and severity classification (i.e., high, medium, low, and normal). For accident image encryption, the multi-key homomorphic encryption (*MKHE*) technique with lion swarm optimization (LSO)-based optimal key generation procedure is involved. In addition, the PPIE-ODLASC approach involves YOLO-v5 object detector to identify the region of interest (ROI) in the accident images. Moreover, the accident severity classification module encompasses Xception feature extractor, bidirectional gated recurrent unit (BiGRU) classification, and Bayesian optimization (BO)-based hyperparameter tuning. The experimental validation of the proposed PPIE-ODLASC algorithm is tested utilizing accident images and the outcomes are examined in terms of many measures. The comparative examination revealed that the PPIE-ODLASC technique showed an enhanced performance of 57.68 dB over other existing models.

## 1. Introduction

Owing to the increase in motorization and population, the number of traffic accidents and their victims seems to be increasing globally [1]. Complicated traffic situations and random events pose a hazard to the safety of drivers, passengers, and pedestrians. Increasing populations and numbers of cars have made traffic accidents a major problem for transportation security. Insurance, medical, and monetary costs all go up when accidents occur on the road. Diverse factors included in traffic accidents have a significant impact on each other, consequently making it tough to individually take any of the parameters while describing the severity of traffic accidents. In the field of traffic safety research, the growth of reliable methods for predicting and classifying crash injury severity, which relies upon numerous explanatory variables, was a key factor [2]. A mechanism for accident management serves a significant role in emergency systems and traffic control. In such structures, data from diverse sources is gathered for supporting injured people [3].

The photographs and measurements taken at the scene of the accident are the most crucial pieces of evidence in cases of accidents. The data collected at the scene of an accident is corrected by police or investigators. There should be no room for error in accident investigations if police and investigators know exactly what they will be using the photos they take at the scene for. It is more efficient to plan out a series of high-quality images rather than taking a dozen random shots. Accident analysis relies heavily on having access to high-quality images of the incidents.

One crucial data source in accidents is image source. Portable or fixed cameras may capture such images, but the latter is very effective. Such digital images generally have a wealth of personally delicate data. When the data is analyzed and collected by attackers, unmeasurable losses will happen along with the leak of personal privacy [4]. The privacy protection of images frequently depends on methods such as privacy encryption, k-anonymity, and access control. Several perceptual encrypted techniques were modelled to generate images without visual data according to the visual data-protection system, but data theory-related encryption (AES and RSA) creates ciphertext [5]. Perceptual encryption intended at generating images without visual data on plain images based on a visual data-protection system as visual data involves private data such as personally identifiable information, time, and place [6].

Conversely, there are several authors on analyzing accidents. Various image-processing approaches were advanced to get a real-time mechanism to assist the accident [7]. Crash severity methods may forecast severity that may be anticipated to occur for a crash that aids clinics in offering proper health care as soon as possible [8]. Moreover, research on crash injury severity even aids superior understanding of what factors contributed to injury severity once a crash occurred, which will help improve road safety and reduce crash severity. Crash severity was generally measured by numerous discrete classes of possible injury, fatal, incapacitating injury, property damage only, and non-incapacitating injury [9].

Because of improvements in processing power and technologies, deep-learning (DL) models have achieved excellent performance in a number of domains, including autonomous vehicle systems. Now that neural networks (NN) have matured into a potent tool for discovering intricate patterns in high-dimensional datasets and delivering on-target predictions, they may now be relied upon to make accurate and trustworthy forecasts in ordinal data. Some of these techniques implement ML techniques such as artificial neural network (ANN). With the help of pooling layers, the hidden features can be derived [10]. Generally, the output of the final pooling layer was implemented for the purposes of regression and classification.

Accident site photos and measurements are the most important evidence. Attackers will steal data and breach personal privacy, causing untold costs. The massive number of images commonly employed poses a significant challenge to privacy preservation, and image encryption can be used to accomplish cloud storage [11] and secure image transmission in the network; moreover, an automated deep-learning (DL)-based accident severity classification is needed.

The novelty of this paper includes:

- This article presents a novel Privacy Preserving Image Encryption with Optimal Deep-Learning-based Accident Severity Classification (PPIE-ODLASC) model. The goal of the presented PPIE-ODLASC technique is to accomplish secure image transmission via encryption and accident severity classification (i.e., high, medium, low, and normal).
- For accident image encryption, multi-key homomorphic encryption (*MKHE*) technique with lion swarm optimization (LSO)-based optimal key generation process is involved.
- In addition, the PPIE-ODLASC algorithm involves YOLO-v5 object detector to identify the region of interest (ROI) in the accident images. Moreover, the accident severity classification module encompasses Xception feature extractor, bidirectional gated recurrent unit (BiGRU) classification, and Bayesian optimization (BO)-based hyperparameter tuning.
- The experimental validation of PPIE-ODLASC technique is tested using accident images and the results are investigated in terms of several measures.

The rest of the paper is organized as follows. Section 2 provides a detailed review of existing models and Section 3 elaborates the proposed algorithm. Then, Section 4 shows experimental validation and Section 5 draws the concluding remarks of the study.

## 2. Literature Review

Boulila et al. [12] advises a hybrid PPDL method for object classification. This study aims to improve the encryption of satellite images while guaranteeing a higher object classifier accuracy and good runtime. The technique projected to encrypt the image is preserved by the public keys of somewhat homomorphic encryption and Paillier homomorphic encryption. Chuman and Kiya [13] developed a learnable image encryption technique for privacy-preserving DNN. The presented technique is performed based on block scrambling utilized along with data augmentation methods, namely grid mask, random cropping, and horizontal flip. The usage of block scrambling improves the robustness against many attacks; on the other hand, combined with data augmentation, it allows the preservation of a higher classifier accuracy while using encrypted images.

He et al. [14] developed a CryptoEyes to overcome the problems of privacy-preserving classifier on encrypted images. The study presents a 2-stream convolution network structure for the classifier of encrypted images to capture the contour of the encrypted image, thereby considerably increasing the accuracy of the classification. Shen et al. [15] developed a secure SVM that is a privacy-preserving SVM training system over blockchain (BC)-based encrypted IoT information. The author utilizes the BC technique to construct reliable and secured data sharing platforms amongst various data providers, whereas an IoT information is encrypted and recorded on the distributed ledger. Ito et al. [16] designed a transformation system to generate visually protected images for privacy-preserving DNN. However, the presented technique allows us to preserve the image classification performance and strongly protects visual information.

The authors in [17] resolve the challenges by designing Secure DL, a privacy-preserving image detection technique for encrypted dataset over cloud. The presented block-based image encryption system is well-developed for protecting the image’s visual data. The presented technique is demonstrated to be secure from a probabilistic perspective, and with different cryptographic attacks. Ahmad and Shin [18] present an effective pixel-based encryption technique. The technique gives a basic level of privacy while maintaining the inherent property of the original images, thus allowing DL application in the encryption field. The author has utilized logistic maps for the lower computation requirement. Furthermore, in order to compensate for any ineffectiveness due to the logistic maps, the author uses a second key for shuffling the sequence.

Li et al. [19] proposed a new FL into autonomous driving for preserving privacy of the vehicle by sharing the model training parameter through MEC server and keeping original information in a local vehicle. Salem et al. [20] introduce DeepZeroID: a multiple-party biometric verification and privacy-preserving cloud-based technique which makes use of homomorphic encryption. Training on sensitive biometric data is eliminated with the help of transfer learning, and one pre-trained DNN is exploited as the feature extractor. By proposing an exhaustive search algorithm, these feature extractors are employed on the processes of liveness detection and biometric authentication. Song et al. [21] present a novel technique that constructs an effective module without sharing sensitive information between the source and target domain. The target domain benefit from the label-rich source domain without exposing its private information. Zhao et al. [22] developed a BC based privacy-preserving software updating protocol that delivers reliable and secure updates with an incentive model while protecting the privacy of the user. Ibarrondo and Önen [23] analyze the Batch Normalization (BN) layer: a modern layer that addresses internal covariance shift, which was demonstrated to be highly effective in improving the performance of the deep neural network. The study aims at reformulating BN that leads to a modest reduction on the number of operations in order to be compatible with the usage of FHE.

Despite the ML and DL algorithms existing in the early research, it is still necessary to optimize the privacy and accident severity classification performance. Simultaneously, various hyperparameters have a crucial effect on the effectiveness of the CNN algorithm. In particular, the hyperparameters including learning rate selection, epoch count, and batch size are necessary to attain superior outcomes. Meanwhile, the trial-and-error algorithm for hyperparameter tuning is an erroneous and challenging task; in the proposed method, the BOA algorithm was used for the parameter selection of the BiLSTM module.

## 3. The Proposed Model

**Figure 1:**
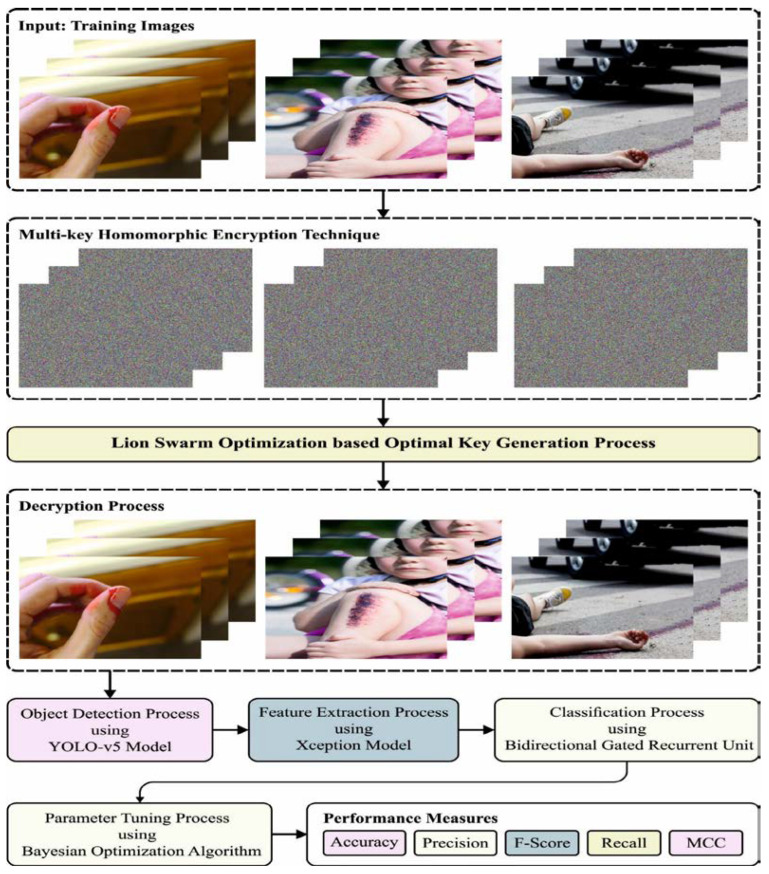
Block diagram of PPIE-ODLASC system.

In this article, we developed a novel PPIE-ODLASC system for privacy and accident severity classification process. In the presented PPIE-ODLASC technique, two major processes are involved, namely encryption and severity classification (i.e., high, medium, low, and normal). At the first level, the accident images are encrypted by the *MKHE* technique with the LSO algorithm, and the encrypted images are transmitted to the received. At the receiving end, the decryption process takes place, and then the accident severity classification process is performed. Figure 1 demonstrates the overall block diagram of the PPIE-ODLASC approach. The detailed working of these processes is deliberated in the following sections.

### 3.1. Image Encryption Module

In this study, the *MKHE* technique is applied to encrypt the accident images. An *MKHE* is a cryptosystem that allows one to evaluate an arithmetic circuit on cipher images, perhaps encrypting in multiple keys. Consider that M remain the message space with arithmetical structure [24]. Assume that each contributing party has a reference to their confidential and public keys. A multi-key cipher image indirectly has an arranged set $T=\{id_{1},\ldots ,id_{k}\}$ related to the reference. For example, a fresh cipher image ct←*MKHE*. $Enc(\mu ;pk_{id})$ is equal to single-element set T={id}; however, the size of references fixed attains better than the calculation among cipher image in party development.

- Setup: pp←MKHE.$Setup(1^{\lambda })$. Proceed with the secure parameters as input and return the public parameterization. Consider that other techniques indirectly get pp as an input.
- Key Generation: (sk,pk)←MKHE.KeyGen(pp). Resulting in a pair of public and confidential keys.
- Encryption: ct←<KHE.Enc(μ;pk). Encrypt a plain image μ∈M and resultant a cipher-image $ct\in \{0,1{\}}^{*}.$
- Decryption: μ←MKHE.$Dec(\bar{ct};\{sk_{id}{\}}_{id\in T})$. For providing a $\bar{ct}$ cipher image with equal order of confidential key, outcome a plain image μ.

The Homomorphic estimation can be described by using Equation (1):

(1) $$\bar{ct}\leftarrow MKHE.Eva1(C,(\bar{ct},\ldots ,{\bar{ct}}_{l}),\left\{pk_{id}{\}}_{id\in T}\right).$$

To provide a C circuit, the equal group of public keys $\{pk_{id}{\}}_{id\in T}$ and a tuple of multi-key cipher-image $\left(\bar{ct},\ldots ,{\bar{ct}}_{l}\right)$ results in a cipher-image $\bar{ct}$. Its reference set is $T=T_{1}\cup \cdots \cup T_{\ell }$ of reference sets = $T_{j}$ of input cipher-image ${\bar{ct}}_{j}$ for 1≤j≤ℓ.

Semantic Security. For two communications $\mu_{0},\mu_{1}\in M$, the distribution {MKHE.Enc ($\mu_{i}$; pk)} for i=0,1 might be undistinguishable while pp←MKHE.$Setup(1^{\lambda })$ and (sk,pk)←MKHE.KeyGen(pp). Compactness and Correctness. An *MKHE* method was compact when the size of cipher images associated with k party is constrained by the poly (λ,k) to set a polynomial poly. Where 1≤j≤ℓ, consider $ct_{j}$ as a cipher image (with $T_{j}$ reference set) as *MKHE*. Considering $C:M^{\ell }\to M$ as the circuit and $\bar{ct}\leftarrow MKHE.Eval(C,(\bar{ct},\ldots ,\bar{ct}),\{pk_{id}{\}}_{id\in T})$ for $T=T_{1}\cup \cdots \cup T_{\ell }$, then,

(2) $$MKHE.Dec(\bar{ct},\left\{sk_{id}{\}}_{id\in T}\right)=C\left(\mu_{1},\ldots ,\mu_{\ell }\right).$$

To optimally select the keys for the *MKHE* technique, the LSO algorithm is exploited. The lion swarm race can be primarily classified into three classes for resolving the global optimization problems of the objective function using the LSO technique: Young Lion, Lion King, and Lioness [25]. They have dissimilar social behaviors. The lioness and lion king are the adult lions, and might affect the difference in convergence speed and the algorithm population size; for maintaining the effects of the algorithm, the proportion of young lion ranges within 0.5 and 1, and the proportion of adult lion τ usually lesser than 0.5. The location of lion king was given in the following:

(3) $$X_{i}^{t+1}=g^{t}(1+\gamma \|P_{i}^{t}-g^{t})$$

where t characterizes the present number of iterations, $X_{i}^{r+1}$ signifies the new position made after the update, $g^{t}$ is an optimum location of t-generation, γ represent the uniformly distributed N(0,1) random number, and $P_{i}^{t}$ is the past optimum position of the i-th lion in t generation population. They cooperate among themselves during hunting, which provides better food to the lion king, and are also accountable to lead the cubs to learn how to hunt; it can be formulated as follows:

(4) $$X_{i}^{t+1}=\frac{\left(P_{i}^{t}+p_{c}^{t}\right)}{2}\left(1+\alpha_{f}\gamma \right)$$

where $X_{i}^{t+1}$ specifies the position of the lioness afterward the update, $P_{c}^{t}$ is the better location in the history of choosing a lioness randomly for cooperating with hunting in t generation population, γ represent the uniformly distributed N(0,1) random number, and $\alpha_{f}$ is a step control factor. The formula for updating the location of the lioness can be given in the following:

(5) $$\alpha_{f}=step\cdot exp(-30t/t_{max}{)}^{10}$$

where step=0.1(H−L) is the maximal moving step of the lioness. Let L and H be the lower and upper boundaries of lion group space correspondingly. $t_{max}$ is a maximal number of iterations.

The young lion has three major behaviors: (1) once the cubs are full, it learns to hunt with the lioness. (2) As an adult, it is evicted from the territory by the lion king and confronted the location of the lion king suffering afterward. (3) If it is hungry, it will eat nearer the lion king. The updated location of the young lions is given as:

(6) $$X_{i}^{r+1}=\left\{\begin{array}{cc} \frac{g^{t}+P_{i}^{t}}{2}\left(1+\alpha_{c}\gamma \right), & q\leq \frac{1}{3}\\ \frac{P_{m}^{t}+P_{i}^{t}}{2}\left(1+\alpha_{c}\gamma \right), & \frac{1}{3}\leq q<\frac{2}{13}\\ \frac{g^{t^{\prime }}+P_{i}^{t}}{2}\left(1+\alpha_{c}\gamma \right), & \frac{2}{3}\leq q<1 \end{array}\right.$$

$X_{i}^{r+1}$ where $X_{i}^{t+1}$ is the position of the young lion, $P_{m}^{t}$ is the better location at t-th generation while the young lion follows the female lion to learn hunting, $\alpha_{c}$ is a step control factor, $\alpha_{c}=step*\left(1-\frac{t}{t_{max}}\right)\cdot g^{t^{\prime }}$ adopt the concept of elite reverse learning that implies the expelled lion cubs are farther from the lion king’s location, and $g^{t^{\prime }}=H+L-g^{t}.$ q refers to a probability factor, a uniformly distributed random integer U(0,1).

The LSO technique proposes deriving the main function depending on the fitness function (FF). The main purpose of the LSO technique is to propose a new image encrypt system with minimized error (MSE) and maximized PNSR. It can be measured as:

(7) $$F=\left\{min\left(MSE\right),max\left(PSNR\right)\right\}.$$

The preferred minimization and maximization values can be achieved with utilization of the LSO system.

### 3.2. Accident Severity Classification Model

In this work, the automated severity classification module comprises different sub processes, namely YOLO-v5 based RoI detection, Xception feature extraction, BiGRU classification, and BO-based hyperparameter tuning.

#### 3.2.1. Accident Region Detection Using YOLO-v5

In the field of artificial intelligence, a convolutional neural network (CNN) is a type of network that is optimized for processing input with a grid-like architecture, such as an image. An electronic photograph is a binary representation of visual information. Semantic segmentation, object detection, fake image identification [26], and image captioning [27] are just a few examples of areas where convolutional neural networks (CNNs) have seen significant advancements in recent years thanks to the explosion of deep learning. With a CNN-LSTM model, features are extracted from input data using CNN layers, while sequence prediction is accomplished using LSTM layers. In order for a neural network to function properly, it needs to be able to store sequence information in both forward and backward directions, a process known as bidirectional long-short term memory (bi-lstm) (past to future). A bi-lstm is distinct from a standard LSTM since its input goes in both directions. Word classification in a text could be another application of bidirectional LSTM. They are more equipped to categorize the word because they can understand its history and its future.

To identify the RoI in the accident images, the YOLO-v5 model is used. YOLOv5 is the most developed object detection technique obtainable. It is a new CNN which performs object detection in real-time with maximum accuracy [28]. This technique utilizes a single NN for processing the whole picture; afterwards, it divides it into parts and forecasts bounding boxes and probability to all the components. These bounding boxes can be weighted by expected possibility. This technique “just looks once” at the image from the sense which it generates forecasts then forwards propagating run with NN. Then, it delivers identified items after non-max suppression.

- Backbone: Backbone has frequently been utilized for extracting the main features in input images. CSP (Cross Stage Partial Network) is utilized as the backbone in YOLOv5 for extracting rich suitable features in an input image.
- Neck: The Neck model was frequently utilized for creating feature pyramids. The feature pyramids aid methods in effective generalizations once it derives to object scaling. It supports the detection of similar objects in several scales and sizes. The feature pyramids can be quite useful in supporting methods for performing effectually on earlier unseen data. Other methods such as PANet, FPN, and BiFPN utilize several sorts of feature pyramid methods. PANet was utilized as a neck from YOLOv5 for obtaining feature pyramid.
- Head: A typical Head was frequently accountable for the last detection stage. It utilizes anchor boxes for constructing last outcome vectors with class probability, objectiveness score, and bounding box.

#### 3.2.2. Xception Based Feature Extraction

At this stage, the features involved in the RoI are extracted by the Xception model. For effective feature extraction, the Xception architecture was introduced to extract feature vectors [29]. Initially, a pretrained Xception network model is selected named Inception. It is a type of deep-CNN architecture that contains a total depth of 71 layers. It is a modified version of Inception-V3 architecture that has surpassed ResNet, Inception-V3, and VGG16 in classification tasks. It encompasses a revised form of depth wise separable convolutional and max-pooling layers, each related as a ResNet. The architecture of Xception consists of: middle flow, exit flow, and entry flow. The input images are passed over the entry flow, following a middle flow, i.e., repeated eight times, and finally, it is passed over the exit flow for data classification. Finetuning can be performed on the exit and middle flow of Xception architecture. The separable convolution layer in the middle flow is reformed after the exit flow and the weight is upgraded to extract relevant features. Following the global average pooling, the extracted features are fed through the topmost model correspondingly comprising four fully connected layers with 256, 128, 1024, and 512 units, each containing an output layer, and ReLU activation is accustomed to data classification.

#### 3.2.3. Severity Classification Using Optimal BiGRU Model

For classification of accident severity into multiple classes, the BiGRU model is exploited in this work. Comparable with LSTM, GRU can be presented for tackling the gradient vanishing problem current in RNNs and studying the long-term dependency from the long sequence applications with internal gating approach [30]. A GRU cell comprises reset gate $r_{n}$ and update gate $z_{n}$. The activation of gates from the GRU was dependent upon presenting input and prior output. The internal infrastructure of the GRU cell in which $h_{n}$ and $x_{n}$ refers to the hidden layer and input vector from the time slice n, and $h_{n}^{\prime }$ implies the candidate of hidden state. For parts n, the reset gate $r_{n}$ determines preceding data has been required for forget and the updating gate $z_{n}$ mechanism upgrading the hidden state with the current EEG data.

(8) $$r_{n}=\sigma \left(W_{r}\cdot \left[h_{n-1},x_{n}\right]\right)$$

(9) $$z_{n}=\sigma \left(W_{z}\cdot \left[h_{n-1},x_{n}\right]\right)$$

(10) $$h_{n}^{\prime }=tanh\left(W_{h^{\prime }}\cdot \left[r_{n}*h_{n-1},x_{n}\right]\right)$$

(11) $$h_{n}=\left(1-z_{n}\right)*h_{n-1}+z_{n}*h_{n}^{\prime }$$

In the aforementioned equation, tanh(·) and σ(·) refer to the hyperbolic tangent and sigmoid functions. · and ∗ symbol implies the matrix multiplication and Hadamard product; furthermore, [] stands for the concatenation of 2 vectors. $W_{z}$, $W_{r},$ and $W_{h^{\prime }}$ signifies the weighted matrix learned by GRU network trained.

Finally, the BO algorithm is used for the optimal hyperparameter adjustment of the BiGRU model. The proposed method is based on the assembly of heuristic approach, whereupon numerous objective tasks was distributed to the objective of concern from the input space [31].

(12) $$D={\left\{\left(a_{x},b_{x}\right)\right\}}_{x=1}^{N}$$

In Equation (12), N refers to the total amount of annotations of the input objective set. A proxy optimization was performed by continuing the BO algorithm to decide the next input. The function used in BO is distributed by means of GPs as a result of systematic, flexible, and ambiguous properties. Thus, BO is utilized to overcome minimization complications as follows:

(13) $$y^{*}=\underset{y\in X^{g(y)}}{argmin}$$

From the expression, X is a dense subset of $R^{K}$. To meta-parameter of substitute method, consider borderline analytical variance of the heuristic model as $\sigma^{2}(y,\Theta )=\Sigma (y,y;\Theta )$ and $\mu \left(y;D,\Theta \right),$ which characterizes the analytical mean and is defined by:

(14) $$\gamma \left(y\right)=\frac{g\left(y_{BEST}\right)-\mu \left(y,D,\Theta \right)}{\sigma \left(y,D,\Theta \right)}$$

In Equation (14), $g(y_{BEST})$ signifies the minimal perceived value and it can be demonstrated below:

(15) $$\alpha_{FI}\left(y,D,\Theta \right)=\sigma \left(y,D,\Theta \right)\cdot \left[\gamma \left(y\right)\Phi \left(\gamma \left(y\right)\right)+M\left(\gamma \left(y\right),0,1\right)\right]$$

In Equation (15), Φ is a cumulative function and M(0,1) is a density of common standard. After the training on the diseased cropped region, the newly trained model is obtained that is used for the feature extraction.

## 4. Experimental Validation

The proposed technique is simulated by means of the Python 3.6.5 tool. The proposed model is experimented on GeForce 1050Ti 4 GB, PC i5-8600k, 16 GB RAM, 1 TB HDD, and 250 GB SSD. The parameter settings are as follows: dropout: 0.5, learning rate: 0.01, activation: ReLU, batch size: 5, and epoch count: 50. The encryption performance of the proposed model is investigated using different measures such as mean square error (MSE), PSNR, structural similarity (SSIM), and root mean square error (RMSE). Next, accuracy, precision, recall, F-score, and Mathew Correlation Coefficient (MCC) can examine the classification performance.

**Figure 2:**
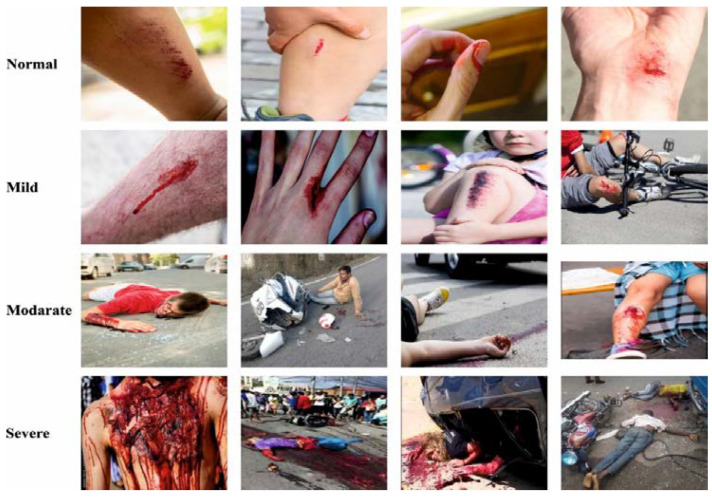
Sample Images of Multiclass.

In this study, we examined the performance of the PPIE-ODLASC model using a set of accident images with four classes. For training purposes, we used the CADP dataset [32], which contains 1416 video segments composed from YouTube, with 205 video segments having full spatio-temporal annotations. For testing purposes, we used our own dataset collected from a real-time environment. It comprises 20,000 samples with four classes (normal, low, medium, and high) as represented in Table 1. Figure 2 defines the sample images of multiclass.

**Figure 3:**
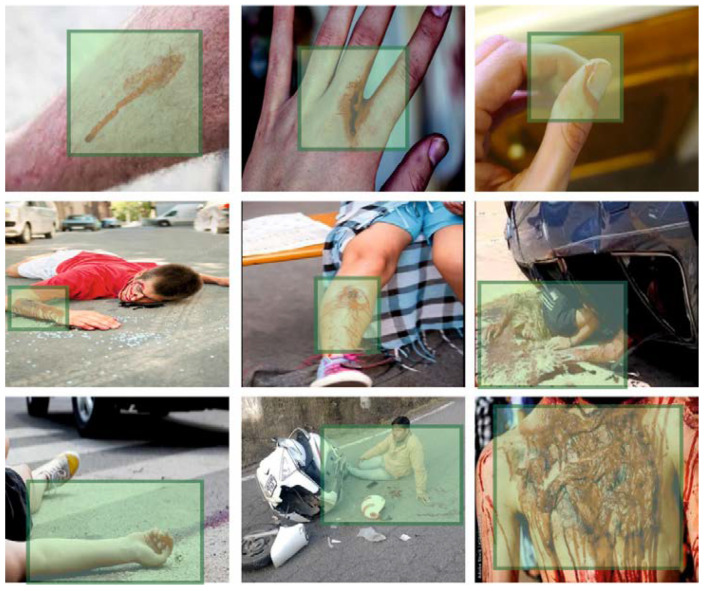
ROI Extraction.

Figure 3 shows the RoI extracted by the PPIE-ODLASC approach on the applied sample images. The result indicates that the PPIE-ODLASC technique has effectually extracted the RoI on all images.

**Figure 4:**
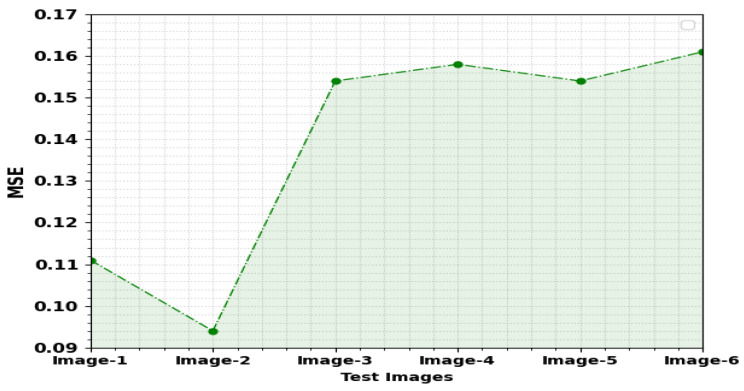
Result analysis of the PPIE-ODLASC system with distinct images.

Table 2 and Figure 4 report the outcomes of the PPIE-ODLASC approach on image encryption process. The outcome stated that the PPIE-ODLASC approach has encrypted the images proficiently. For instance, on image1, the PPIE-ODLASC system has obtained an MSE of 0.1110, RMSE of 0.3332, PSNR of 57.68 dB, and SSIM of 99.81%. Meanwhile, in image3, the PPIE-ODLASC method has reached an MSE of 0.1540, RMSE of 0.3924, PSNR of 56.26 dB, and SSIM of 99.95%. Eventually, on image6, the PPIE-ODLASC technique gained an MSE of 0.1610, RMSE of 0.4012, PSNR of 56.06 dB, and SSIM of 99.87%.

**Figure 5:**
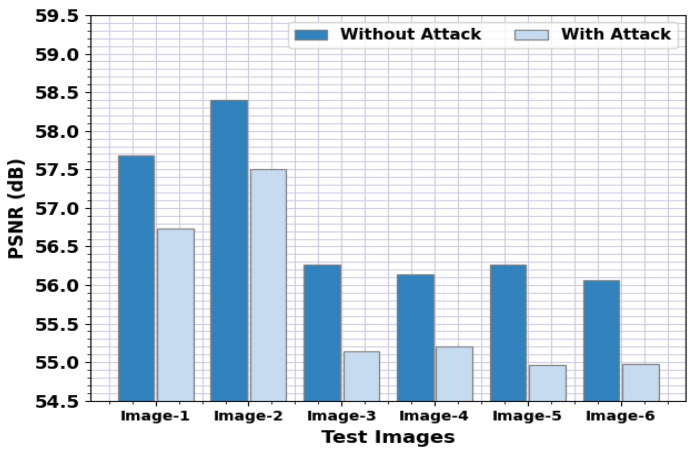
PSNR analysis of the PPIE-ODLASC system under with and without attacks.

Table 3 and Figure 5 represent the PSNR results of the PPIE-ODLASC system with and without attacks. The outcome indicated that the PPIE-ODLASC algorithm has obtained effectual PSNR values under the presence of attack. For sample, in image1, the PPIE-ODLASC approach has obtained a PSNR of 57.68 dB and 56.73 dB for without and with attacks, respectively. Concurrently, on image3, the PPIE-ODLASC method has gained a PSNR of 56.26 dB and 55.14 dB for without and with attacks, correspondingly. Furthermore, in image6, the PPIE-ODLASC model has obtained a PSNR of 56.06 dB and 54.98 dB for without and with attacks, correspondingly.

**Figure 6:**
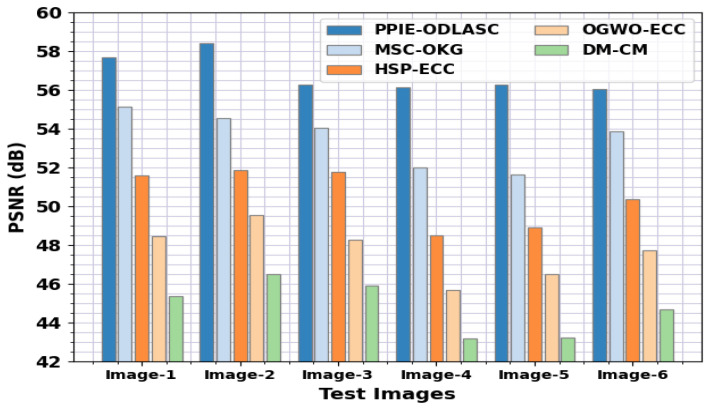
PSNR analysis of the PPIE-ODLASC system under distinct images.

A comparative PSNR study of the PPIE-ODLASC approach with other existing methods on various images is given in Table 4 and Figure 6. The outcome highlighted that the PPIE-ODLASC system reached higher PSNR values. For instance, in image1, the PPIE-ODLASC methodology obtained an improved PSNR of 57.68 dB, while the MSC-OKG, HSP-ECC, OGWO-ECC, and DM-CM models obtained a reduced PSNR of 55.14 dB, 51.60 dB, 48.45 dB, and 45.37 dB, respectively. Similarly, in image 3, the PPIE-ODLASC model reached an improved PSNR of 56.26 dB, while the MSC-OKG, HSP-ECC, OGWO-ECC, and DM-CM [33] models obtained a reduced PSNR of 54.02 dB, 51.77 dB, 48.26 dB, and 45.88 dB, correspondingly. Additionally, in image 6, the PPIE-ODLASC model obtained an improved PSNR of 56.06 dB, while the MSC-OKG, HSP-ECC, OGWO-ECC, and DM-CM models obtained a reduced PSNR of 53.86 dB, 50.36 dB, 47.72 dB, and 44.69 dB, correspondingly.

**Figure 7:**
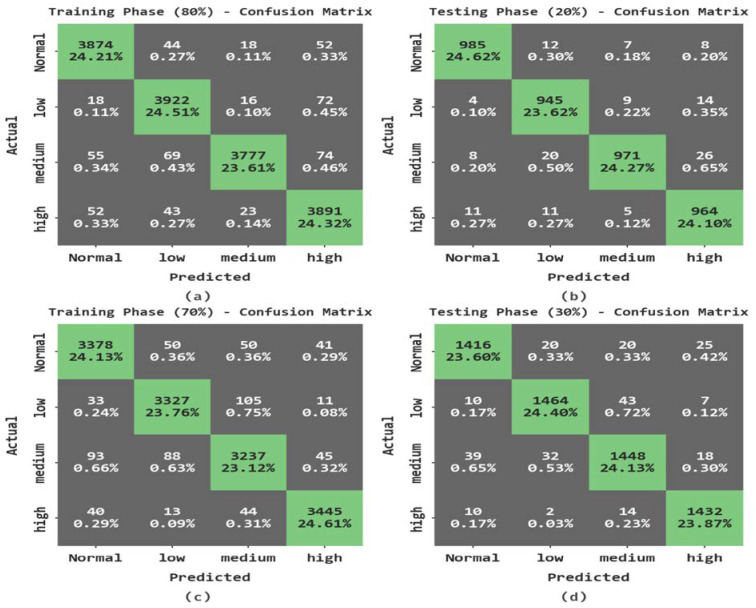
Confusion matrices of the PPIE-ODLASC approach; (**a**,**b**) TR and TS databases of 80:20 and (**c**,**d**) TR and TS databases of 70:30.

The accident severity classification results of the PPIE-ODLASC model in terms of the confusion matrix are shown in Figure 7. The results indicated that the PPIE-ODLASC model has accurately classified different types of severity levels.

Table 5 represents an overall accident severity classification result of the PPIE-ODLASC model under different sizes of TR and TS databases. The experimental results stated that the PPIE-ODLASC model has accurately identified varying levels of severity. For example, with 80% of TR data, the PPIE-ODLASC technique offered an average $accu_{y}$ of 98.32%, $prec_{n}$ of 96.68%, $reca_{l}$ of 96.65%, $F_{score}$ of 96.65%, and MCC of 95.54%. Along with that, with 20% of TS database, the PPIE-ODLASC technique offered an average $accu_{y}$ of 98.31%, $prec_{n}$ of 96.63%, $reca_{l}$ of 96.64%, $F_{score}$ of 96.62%, and MCC of 95.51%. Moreover, with 70% of TR database, the PPIE-ODLASC methodology offered an average $accu_{y}$ of 97.81%, $prec_{n}$ of 95.61%, $reca_{l}$ of 95.61%, $F_{score}$ of 95.61%, and MCC of 94.15.

**Figure 8:**
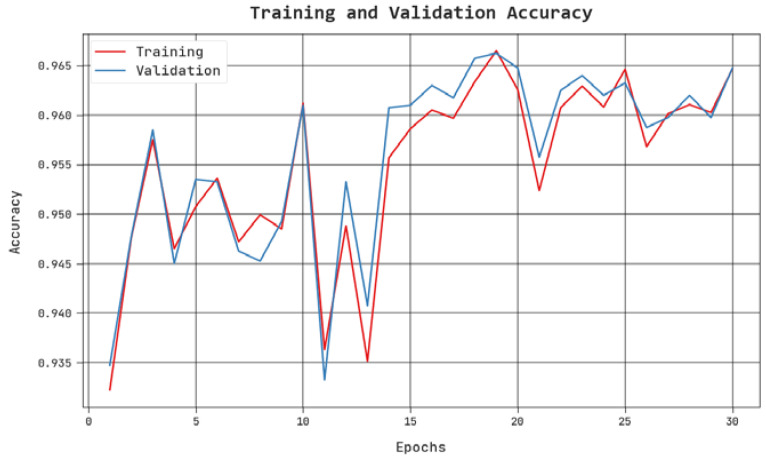
TACC and VACC analysis of PPIE-ODLASC approach.

The TACC and VACC of the PPIE-ODLASC approach are examined on accident severity classification performance in Figure 8. The figure exhibited that the PPIE-ODLASC method has shown improved outcomes with increased values of TACC and VACC. In particular, the PPIE-ODLASC method has reached maximum TACC outcomes.

**Figure 9:**
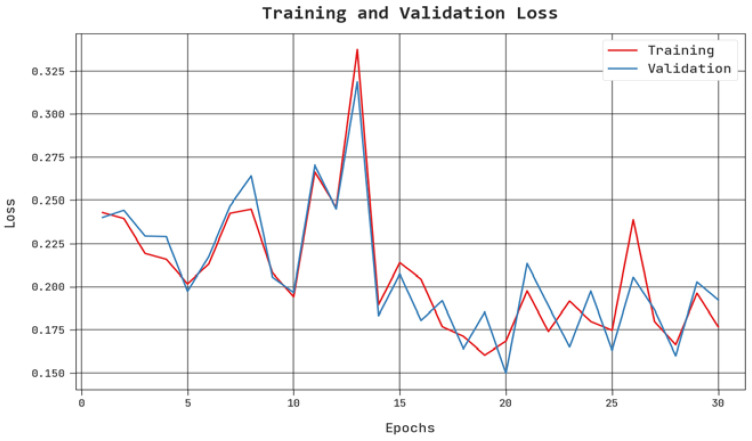
TLS and VLS analysis of the PPIE-ODLASC approach.

The TLS and VLS of the PPIE-ODLASC method are tested on accident severity classification performance in Figure 9. The figure shows that the PPIE-ODLASC approach has revealed better performance with minimal values of TLS and VLS. Notably, the PPIE-ODLASC methodology has resulted in reduced VLS outcomes.

**Figure 10:**
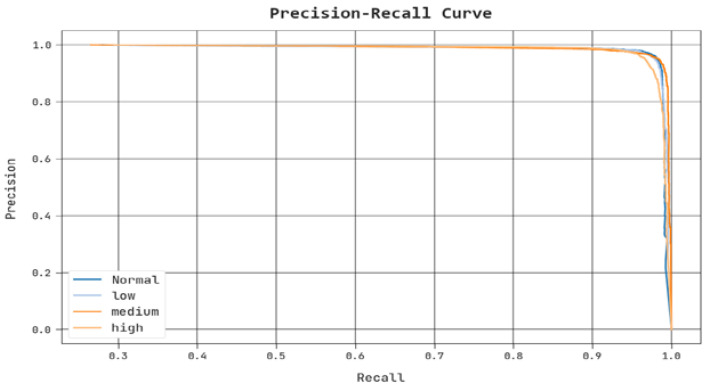
Precision-recall analysis of PPIE-ODLASC methodology.

A clear precision-recall investigation of the PPIE-ODLASC approach under test database is seen in Figure 10. The figure indicated that the PPIE-ODLASC method has superior values of precision-recall values under several classes.

**Figure 11:**
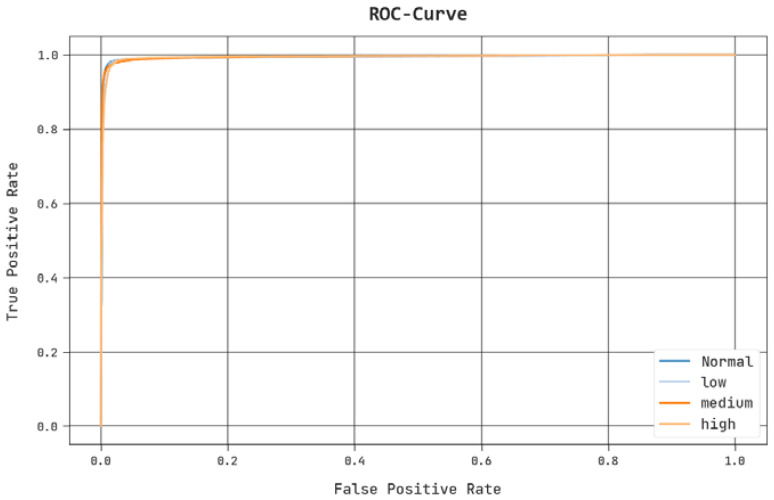
ROC analysis of the PPIE-ODLASC approach.

A brief ROC study of the PPIE-ODLASC method under test database is shown in Figure 11. The result denotes the PPIE-ODLASC algorithm has demonstrated its ability in categorizing distinct classes.

**Figure 12:**
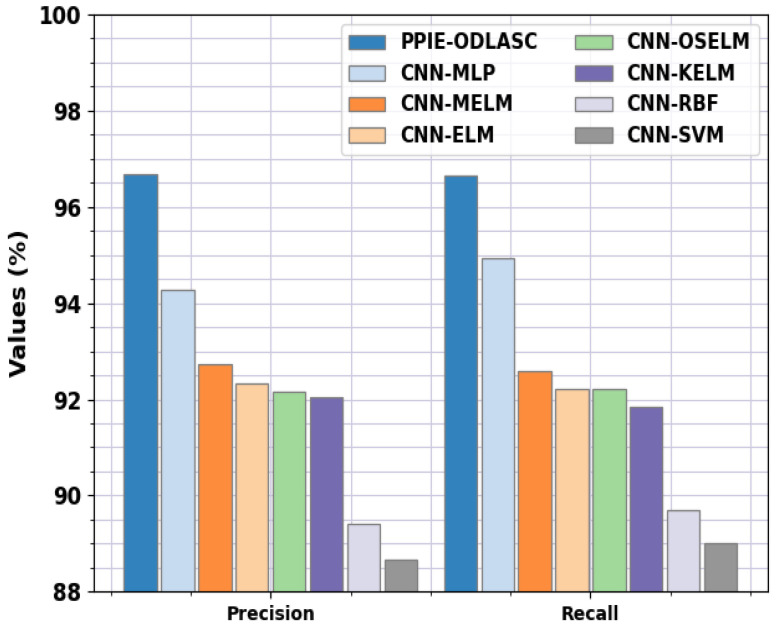
$Prec_{n}$ and $Reca_{l}$ analysis of the PPIE-ODLASC approach with other recent systems.

In Table 6, a detailed comparison study of the PPIE-ODLASC with current DL techniques such as CNN with multilayer perceptron (MLP), CNN with multi-kernel extreme learning machine (MELM), CNN with extreme learning machine (CNN-ELM), CNN with optimal stacked extreme learning machine (CNN-OSELM), CNN with kernel extreme learning machine (CNN-KELM), CNN with radial basis function (CNN-RBF), and CNN with SVM (CNN-SVM) is provided [34]. Figure 12 represents the comparative accident severity classification results of the PPIE-ODLASC model with respect to $prec_{n}$ and $reca_{l}$. The experimental results stated that the PPIE-ODLASC model has gained enhanced performance. Based on $prec_{n}$, the PPIE-ODLASC model has gained increased $prec_{n}$ values of 96.68%, while the CNN-MLP, CNN-MELM, CNN-ELM, CNN-OSELM, CNN-KELM, CNN-RBF, and CNN-SVM models have reported reduced $prec_{n}$ values of 94.28%, 92.73%, 92.33%, 92.16%, 92.05%, 89.40%, and 88.66%, respectively. At the same time, based on $reca_{l}$, the PPIE-ODLASC method has obtained increased $reca_{l}$ values of 96.65%, while the CNN-MLP, CNN-MELM, CNN-ELM, CNN-OSELM, CNN-KELM, CNN-RBF, and CNN-SVM [31] approaches have reported reduced $reca_{l}$ values of 94.94%, 92.60%, 92.22%, 92.22%, 91.84%, 89.70%, and 89%, respectively.

**Figure 13:**
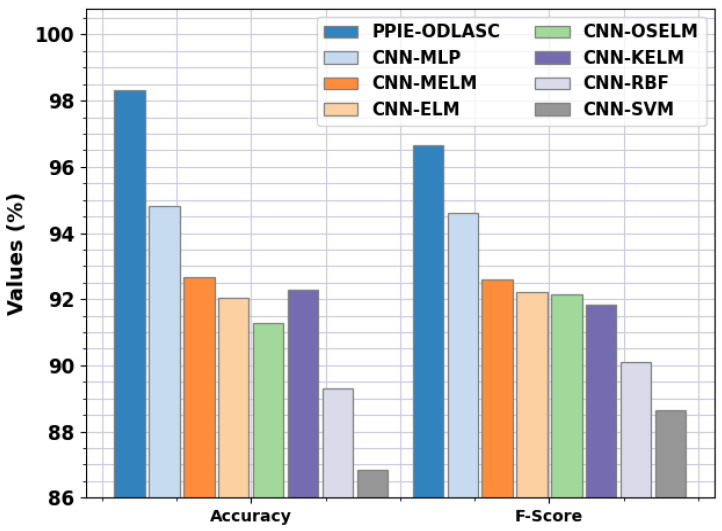
$Accu_{y}$ and $F_{score}$ analysis of the PPIE-ODLASC approach with other recent systems.

Figure 13 represents the comparative accident severity classification results of the PPIE-ODLASC technique in terms of $accu_{y}$ and $F_{score}$. The result shows that the PPIE-ODLASC technique has reached enhanced performance. Based on $accu_{y}$, the PPIE-ODLASC technique has acquired increased $accu_{y}$ values of 98.32%, while the CNN-MLP, CNN-MELM, CNN-ELM, CNN-OSELM, CNN-KELM, CNN-RBF, and CNN-SVM methods have reported reduced $accu_{y}$ values of 94.80%, 92.66%, 92.03%, 91.28%, 92.29%, 89.30%, and 86.83%, respectively.

Simultaneously, based on $F_{score}$, the PPIE-ODLASC technique has gained increased $F_{score}$ values of 96.65%, while the CNN-MLP, CNN-MELM, CNN-ELM, CNN-OSELM, CNN-KELM, CNN-RBF, and CNN-SVM models have reported reduced $F_{score}$ values of 94.60%, 92.60%, 92.20%, 92.13%, 91.84%, 90.10%, and 88.66%, respectively.

**Figure 14:**
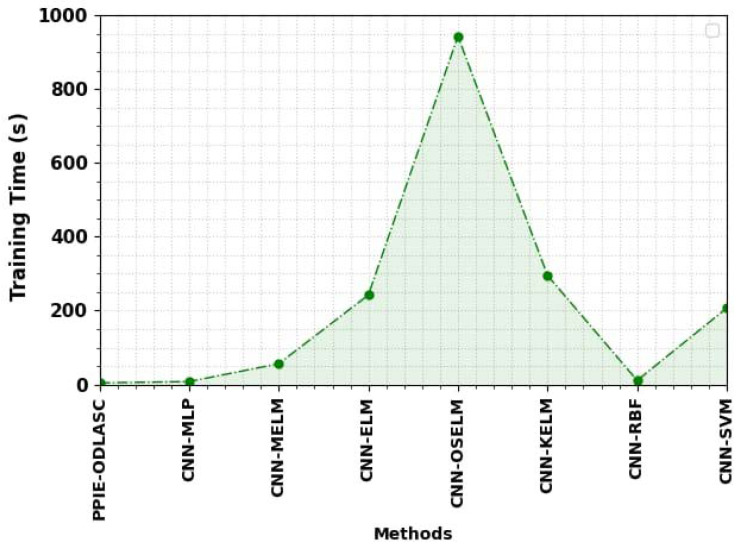
TRT analysis of the PPIE-ODLASC approach with other recent systems.

Finally, a detailed training time (TRT) inspection of the PPIE-ODLASC with other DL methods takes place in Figure 14. The results implied that the PPIE-ODLASC approach has gained better performance with a minimal TRT of 4.39 s. Contrastingly, the CNN-MLP, CNN-MELM, CNN-ELM, CNN-OSELM, CNN-KELM, CNN-RBF, and CNN-SVM models have reported increased TRT of 94.28%, 92.73%, 92.33%, 92.16%, 92.05%, 89.40%, and 88.66%, respectively. The result shows the superior performance of the PPIE-ODLASC approach over other existing techniques.

## 5. Conclusions

In this article, we developed a new PPIE-ODLASC technique for privacy and accident severity classification process. Initially, the PPIE-ODLASC technique encrypted the accident images using LSO with *MKHE* technique, where the design of LSO-based key generation process helps in the maximization of PSNR. Next, the severity classification module comprises YOLO-v5 based RoI detection, BiGRU classification, Xception feature extraction, and BO-based hyperparameter tuning. The experimental validation of the proposed PPIE-ODLASC technique is tested utilizing accident images and the outcomes are examined in terms of many measures. The comparative examination revealed that the PPIE-ODLASC technique has shown superior performance over other existing approaches. Compared with the other methods, the PPIE-ODLASC method’s F score has improved, reaching 96.65%, while the F scores of the CNN-MLP, CNN-MELM, CNN-ELM, CNN-OSELM, CNN-KELM, CNN-RBF, and CNN-SVM models have decreased. In the future, hybrid metaheuristic algorithm can be derived to enhance the performance of the PPIE-ODLASC technique.

## Figures and Tables

**Table 1:** Details of dataset.

Class	No. of Instances
Normal	5000
low	5000
medium	5000
high	5000
**Total number of Instances**	**20,000**

**Table 2:** Result analysis of the PPIE-ODLASC system with various images.

Test Images	MSE	RMSE	PSNR	SSIM (%)
Image1	0.1110	0.3332	57.68	99.81
Image2	0.0940	0.3066	58.40	99.95
Image3	0.1540	0.3924	56.26	99.95
Image4	0.1580	0.3975	56.14	99.86
Image5	0.1540	0.3924	56.26	99.80
Image6	0.1610	0.4012	56.06	99.87

**Table 3:** PSNR analysis of the PPIE-ODLASC system under with and without attacks.

Test Images	Without Attack	With Attack
Image-1	57.68	56.73
Image-2	58.40	57.50
Image-3	56.26	55.14
Image-4	56.14	55.21
Image-5	56.26	54.96
Image-6	56.06	54.98

**Table 4:** PSNR analysis of the PPIE-ODLASC system with other approaches under different images.

PSNR (dB)					
Test Images	PPIE-ODLASC	MSC-OKG	HSP-ECC	OGWO-ECC	DM-CM
Image-1	57.68	55.14	51.60	48.45	45.37
Image-2	58.40	54.54	51.84	49.56	46.47
Image-3	56.26	54.02	51.77	48.26	45.88
Image-4	56.14	52.00	48.47	45.69	43.17
Image-5	56.26	51.65	48.89	46.49	43.21
Image-6	56.06	53.86	50.36	47.72	44.69

**Table 5:** Accident severity classification outcome of the PPIE-ODLASC approach with varying measures.

Class	$Accu_{y}$	$Prec_{n}$	$Reca_{l}$	$F_{score}$	MCC
**Training Phase (80%)**					
Normal	98.51	96.87	97.14	97.01	96.01
low	98.36	96.17	97.37	96.77	95.67
medium	98.41	98.51	95.02	96.73	95.71
high	98.02	95.16	97.06	96.10	94.78
**Average**	**98.32**	**96.68**	**96.65**	**96.65**	**95.54**
**Testing Phase (20%)**					
Normal	98.75	97.72	97.33	97.52	96.69
low	98.25	95.65	97.22	96.43	95.28
medium	98.12	97.88	94.73	96.28	95.05
high	98.12	95.26	97.28	96.26	95.01
**Average**	**98.31**	**96.63**	**96.64**	**96.62**	**95.51**
**Training Phase (70%)**					
Normal	97.81	95.32	95.99	95.65	94.19
low	97.86	95.66	95.71	95.69	94.26
medium	96.96	94.21	93.47	93.84	91.83
high	98.61	97.26	97.26	97.26	96.33
**Average**	**97.81**	**95.61**	**95.61**	**95.61**	**94.15**
**Testing Phase (30%)**					
Normal	97.93	96.00	95.61	95.81	94.43
low	98.10	96.44	96.06	96.25	94.98
medium	97.23	94.95	94.21	94.58	92.72
high	98.73	96.63	98.22	97.41	96.58
**Average**	**98.00**	**96.00**	**96.03**	**96.01**	**94.68**

**Table 6:** Comparative analysis of PPIE-ODLASC with other recent systems.

Methods	$Prec_{n}$	$Reca_{l}$	$F_{score}$	$Accu_{y}$	Training Time (s)
PPIE-ODLASC	96.68	96.65	96.65	98.32	04.39
CNN-MLP	94.28	94.94	94.60	94.80	07.87
CNN-MELM	92.73	92.60	92.60	92.66	56.16
CNN-ELM	92.33	92.22	92.20	92.03	242.18
CNN-OSELM	92.16	92.22	92.13	91.28	942.86
CNN-KELM	92.05	91.84	91.84	92.29	295.14
CNN-RBF	89.40	89.70	90.10	89.30	10.94
CNN-SVM	88.66	89.00	88.66	86.83	206.74

## Data Availability

Not applicable.
